# Metrological Challenges in Collaborative Sensing: Applicability of Digital Calibration Certificates

**DOI:** 10.3390/s20174730

**Published:** 2020-08-21

**Authors:** Tuukka Mustapää, Pekka Nikander, Daniel Hutzschenreuter, Raine Viitala

**Affiliations:** 1 School of Engineering, Aalto University, 02150 Espoo, Finland; raine.viitala@aalto.fi; 2 School of Electrical Engineering, Aalto University, 02150 Espoo, Finland; pekka.nikander@aalto.fi; 3 Physikalisch-Technische Bundesanstalt (PTB), Division 1–Mechanics and Acoustics, 38116 Braunschweig, Germany; daniel.hutzschenreuter@ptb.de

**Keywords:** IoT-communication, sensor networks, smart agents, metrology, digital calibration certificate, traceability

## Abstract

IoT systems based on collaborative sensor networks are becoming increasingly common in various industries owing to the increased availability of low-cost sensors. The quality of the data provided by these sensors may be unknown. For these reasons, advanced data processing and sensor network self-calibration methods have become popular research topics. In terms of metrology, the self-calibration methods lack the traceability to the established measurement standards of National Metrology Institutes (NMIs) through an unbroken chain-link of calibration. This problem can be solved by the ongoing digitalization of the metrology infrastructure. We propose a conceptual solution based on Digital Calibration Certificates (DCCs), Digital SI (D-SI), and cryptographic digital identifiers, for validation of data quality and trustworthiness. The data that enable validation and traceability can be used to improve analytics, decision-making, and security in industrial applications. We discuss the applicability and benefits of our solutions in a selection of industrial use cases, where collaborative sensing has already been introduced. We present the remaining challenges in the digitization and standardization processes regarding digital metrology and the future work required to address them.

## 1. Introduction

The use of collaborative sensor networks in industrial applications is increasing. As an example, a typical application area has been monitoring environmental conditions and air quality by measuring the concentrations of gases, temperature, humidity, or pressure [1]. Cyber-physical systems are often based on the use of a large number of sensors to collect data and to monitor several parameters, such as vibrations [2].

The use of sensor networks has become more frequent largely because of the increased availability of low-cost sensors [3]. This allows cost efficient deployment of a large number of sensors for collecting data from multiple sources. However, the quality of the data from these types of sensors can vary widely, signifying that post-processing and data fusion are needed, e.g., to filter anomalies.

A stand-alone sensor network does not allow metrological traceability of the measurements. To ensure traceability, instruments need to be calibrated with reference instruments that are traceable all the way to the corresponding national measurement standards [4]. This unbroken link between a measurement instrument and a measurement standard is typically referred to as the calibration chain of an instrument.

Calibrating individual sensors of a sensor network can be complex and costly because of several reasons, such as a large number of sensors that need to be calibrated or difficult accessibility of the locations where the sensors are deployed. Furthermore, if sensors are calibrated in an uncontrolled environment, e.g., their deployment positions, the result of the calibration will be more affected by the environmental conditions [1]. One practice in sensor networks is using a set of selected sensors in the network as references for data fusion and bespoke algorithms. This kind of a practice is referred to as self-calibration.

The main problem with self-calibrating sensors in uncontrolled environments is that the calibration chains of the sensors and thus the traceability of the measurement results can become excessively indistinctive over time, which will lead to increasing measurement uncertainties. Additionally, these kind of calibrations are poorly applicable for compensating errors caused by the aging of the sensors, as it is likely that similar sensors will be affected similarly by aging. This may lead to a situation where all the sensors in the network give systematically erroneous values, for example, due to drifting similarly. This kind of a systematic error in the data cannot be noticed via self-calibration. Hence, the term self-calibration can be misleading, since while it can be used to improve data quality as such, it does not provide the traceability that is essential for trustworthy measurement results.

In this paper, we propose a conceptual solution for improving the measurement traceability in IoT sensor networks. In short, our solution is based on using cryptographic device identifiers, Digital Calibration Certificates (DCCs), and Digital SI (D-SI) data model to enhance the measurement data of IoT sensors with calibration information and thus to improve data trustworthiness by enabling validation of sensor measurement data uncertainty, integrity, and authenticity.

The rest of this paper is organized as follows. In Section 2, we go through the background of this study and discuss the relevant aspects of metrology, collaborative sensing, security in IoT, and digital identifiers. In Section 3, we go through the previous works related to the use of smart agents and determining the measurement uncertainty in sensor networks. In Section 4, we present our conceptual solution for enhancing IoT sensor data with digitally signed DCCs and digital identifiers, and an example architecture for a smart sensor agent system. In Section 5, we discuss a selection of used cases of collaborative sensing that could benefit from the enhanced measurement data provided by our proposed solution. In Section 6, we discuss the remaining challenges and future work related to the topic. Finally, Section 7 provides a conclusion of our work and summarizes this paper.

## 2. Background

Decentralization is currently a big trend, for example, in the development of network-based systems and thereby also in collaborative IoT sensor networks. However, from a metrological point of view, fully decentralized systems can be considered as somewhat problematic, because in metrology the comparability of measurement results is made possible by traceability to measurement standards, which is strongly based on a hierarchical infrastructure.

### 2.1. Metrology and the Importance of the Calibration Process

Metrology is the discipline that covers measurements. Because measurements have a critical role in a large variety of applications, such as quality management and monitoring systems providing the foundation of global trade and industrial collaboration, maintaining the global metrology infrastructure also requires active collaboration. The global infrastructure is maintained by the International Bureau of Weights and Measures (BIPM) and National Metrology Institutes (NMIs), which implement and maintain the national measurement standards [4,5]. The collaboration is based on specific standards and agreements such as the CIPM Mutual Recognition Agreement (MRA) [6].

In addition to the NMIs, National Accreditation Bodies (NABs) oversee accredited calibration laboratories and service providers and verify that the laboratories and service providers fulfill all relevant regulations and standards, such as ISO/IEC 17025 [7].

In the BIPM’s International Vocabulary in Metrology (VIM), metrological traceability is defined as a property of a measurement result whereby the result can be related to a reference through a documented unbroken chain of calibrations [8]. Measurement traceability is based on an established calibration hierarchy. NMIs are at the top of this hierarchy and provide national standards for fundamental physical measurement quantities, like temperature, mass, and electric current, with very small uncertainty. By going down each level of the calibration hierarchy—by each calibration chain link—the measurement uncertainty increases.

BIPM defines calibration as an operation that under specified conditions establishes a relation between the quantity values with measurement uncertainties provided by a measurement standard and corresponding indications, with associated measurement uncertainties, and uses this information to establish a relation for obtaining a measurement result from an indication [8].

Measurement uncertainty is defined in VIM as the parameter characterizing the dispersion of the quantity values being attributed to a measurand based on the information used [8]. The standard measurement uncertainty can be evaluated based on probability distributions either from a probability density function derived from an observed frequency distribution (type A) or subjective probability (type B) [9]. The standard measurement uncertainty can be multiplied by a coverage factor *k* to obtain expanded uncertainty, which is often required in industrial regulations associated with safety or quality management. The factor is usually selected in the range from two to three depending on the coverage probability and level of confidence required.

If the measures and scales are not traced back to measurement standards, i.e., through an unbroken chain of calibration, then it is impossible to quantify the accuracy of measurement values they give. That is, the magnitude of deviations between the value from the measurement and the underlying unknown true quantity value that was intended to be measured cannot be quantified [5,10]. In case of traceability through calibration, the magnitude of deviations is expressed by the measurement uncertainty.

The automotive industry is an illustrative example for the need of the knowledge on the uncertainty of measurement and traceability to unified measurement standards. For quite a long time, the parts used to produce cars have been coming from global supply chains. To successfully assemble the parts, it is crucial that all manufacturers from the supply chains can access the real geometry of their work pieces with the same accuracy of length measures. If problems occur, e.g., manufactured parts are not meeting their specified tolerances, the cause for the problem must be determined to avoid further excessive costs and delays, which can be a laborious process.

### 2.2. Collaborative Sensing

Collaborative sensing has been studied in a variety of applications. One of the major interests behind the use of collaborative sensor networks is that they provide relatively cost-efficient data sources for process optimization and machine learning (ML) applications. Although the use of sensors in the IoT environments has been becoming more and more popular, the metrological traceability of measurements in sensor networks has not been widely studied [3]. However, it is acknowledged that there are problems related to the calibration of sensor networks [1,11,12]. In a review of the current state-of-the-art of low-cost sensor networks, He et al. have described the challenges that the use of low cost sensors cause [12]. The challenges include *measurement faults,* caused by systematic or random sensor bias, and synchronization or *communication faults,* as the capabilities of low-cost sensors are limited. Improving the data quality cost-efficiently is difficult due to the costs and laboriousness of traditional calibration procedures, which require access to the devices and typically cause interruptions in operation [1]. Mainly for these reasons, a lot of effort has been globally put into development of computational self-calibration and data processing methods to improve data quality and sensor network lifetime, e.g., by Kalman filtering and ML [12,13,14,15].

Currently, calibration information can be linked to a sensor by digital data sheets, such as transducer electronic data sheets (TEDS) described in the IEEE 1451 standards, but accredited calibrations themselves are currently only documented on paper or PDF-based calibration certificates [16]. A lack of a standard for machine-readable digital calibration information, which would cover the whole calibration chain from national measurement standards all the way to low-cost sensors, has limited the use of calibration information, largely because of the incompatibility issues between different systems. The lack of machine-readability requires that the calibration data has had to be manually entered into the data management systems, which is excessively complicated for sensor network purposes when the number of sensors is large, which in turn is one of the prerequisites for effective collaborative sensing. Additionally, manual data entry is a possible cause for human errors.

Decentralization and peer-to-peer communication-based self-calibration methods are problematic, if the self-calibration is performed without some “ground truth” nodes that act as a traceable reference. In collaborative sensor networks, it is common that the sensors are self-calibrated to verify that all sensors of a particular network perform similarly and to correct the output of inaccurate sensors by adjusting the transfer function parameters [1]. However, without a reference that is traceable to the national measurement standards, the measurement uncertainty of the network cannot truly be maintained within specific boundaries. For these reasons, the term self-calibration can be misleading if the system does not include traceable reference nodes.

### 2.3. Security

For a long time, computer and communications security was in many respects neglected in IoT, largely because of cost and time-to-market (TTM) concerns. More recently, a lot of attention has been placed on the threat that insecure IoT devices cause to the rest of the Internet [17,18]. That is, since IoT devices typically work unattended by humans and since they are easy to break into, they were and are still being used as platforms for so-called botnet attacks, where a large number of compromised IoT devices are reprogrammed to send forged traffic toward other victims in the Internet, causing the attack targets to clog, misbehave, or crash.

However, from the metrology point of view, the main concern is whether the measurement results are trustworthy. If an IoT device is compromised, clearly anything it does cannot be trusted any more, as the intruder has potentially complete control over the device. Hence, if the measurements are to be trusted, it is of utmost importance to make sure that the individual sensors remain uncompromised. 

There are multiple approaches to secure individual devices. First, the security of the devices themselves can be enhanced through better software development methods, including explicit penetration testing [19,20]. Second, the devices may be separated from the Internet with a firewall or proxy, thereby reducing the attack surface [21]. These proxies or gateways may even include intrusion detection features, intelligently monitoring the behavior of the IoT devices [22]. Third, security may be enhanced by better hardware, i.e., through including into the IoT devices hardware features that allow cryptographic keys to be secured from the IoT application software or even the whole software to be remotely attested [23]. For example, ARM TrustZone has recently became available even in the lowest end 32-bit microcontrollers [24,25].

### 2.4. Digital Identifiers

From the measurements point of view, it is also of utmost interest that the measurement results are not compromised after they leave the IoT device. That is, even if the IoT device itself is completely secured, running its intended software, when the measurement data leave the device through some communications medium, it become vulnerable. These communication threats have been countered by placing the measurement devices into separate, physically protected networks. However, if the results are later communicated over the Internet, they become vulnerable there.

For wireless sensor networks, already the first wireless link is a threat. Furthermore, even if the network itself is protected by cryptographic means, the process of attaching an IoT device to the network for the first time is hard to do in a really secure manner [26,27]. One approach to counter the wireless and attachment vulnerabilities is to assign a cryptographically secure identifier to all IoT devices already before deployment, e.g., at the manufacturing time. If these preconfigured identifiers are then configured at the system side before a device is deployed, already the initial connection between a new device and the rest of the system can be secured [28,29].

One method for applying cryptographically bound device identities is using Secure Device Identities (DevIDs) based on the IEEE 802.1AR-2018 standard [30]. A drawback of such public key identity and signature schemes is that using a public key, or its fingerprint identifier, allows third parties to de-anonymize and track the origin of the data. Where data may comprise personally identifiable information (PII), privacy issues are of concern. To address these issues, the World Wide Web Consortium (W^3^C) has two working groups: Decentralized Identifiers and Verifiable Credentials. This work is based on the concept of Self-Sovereign Identities (SSIs), which have led to the application of Decentralized Identifiers (DIDs) to devices [31,32,33]. More recently, there have been ongoing discussions between the W^3^C Device Identifiers and Web of Things working groups on standardization of device DIDs.

## 3. Related Work

Inadequate data quality is commonly compensated by using a large number of sensors in a system and using post-processing algorithms for enhancing the data by e.g., data fusion, in which the data from multiple sources is combined into one large data set, which then can be analyzed to observe and filter anomalies and faulty measurement data samples. In the European Metrology Programme for Innovation and Research (EMPIR) project Metrology for Factory of the Future (Met4FoF), a platform for simultaneous calibration of several MEMS sensors has been developed to help reduce the cost of calibrating individual sensors [3]. In the Met4FoF project, a concept for using sensor agents for including the sensor specific measurement uncertainty information based on the calibrations was also proposed by Eichstädt et al. [3]. 

The use of smart agents in sensor networks has also been studied in numerous publications. Leppänen et al. [34] describe a smart agent being a device that is capable to act autonomously, possess self-properties, and allow the direct manipulation of the hosting device and its physical components, such as sensors or actuators.

Yong et al. have presented a demonstrator system for using the sensor agents to improve the handling of uncertainty in predictive ML in a cyber-physical manufacturing system (CPMS) [35]. ML has become more and more popular in optimizing operations and processes in manufacturing and quality control as it can effectively be used, for example, in predictive maintenance. As ML algorithms are dependable on the input data quality, the predictive accuracy will be affected by the data source uncertainties. Yong et al. have proposed an architecture that can be used to take the various sources of uncertainty, including the sensors’ measurement uncertainty, into account in the predictions. Salvador Palau et al. have presented an advanced multi-agent system for real-time distributed collaborative prognostics [36]. 

Although multi-agents systems can provide significant benefits, a lack of knowledge or suitable tools can slow down the introduction in practice. Hanga et al. have described potential use of ML and multi-agent systems in oil and gas industry applications [37]. In the oil and gas industry, the use of ML and multi-agent systems are looked into as tools for streamlining processes and cutting costs, but so far their use has been limited by a lack of knowledge, suitable development tools, and standards, investment costs and cautious attitude toward new technologies replacing existing systems.

The EMPIR project SmartCom aims at establishing a basis for a global standard for Digital Calibration Certificates (DCC) and a standardized data model and presentation format for metrological data, Digital SI (D-SI) [38]. Publications describing the DCC and D-SI have been presented by Hackel et al. and Hutzschenreuter et al. [39,40]. 

**Figure 1:**
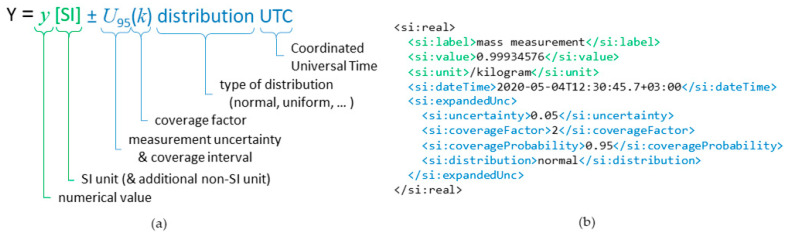
The Digital SI (D-SI) universal data model (**a**) and an example data sample in the XML format (**b**). Minimum requisite information is shown in green and the timestamp and expanded measurement uncertainty in blue.

The concept and original XML schema for the D-SI data model was developed in SmartCom as a solution for unambiguous and machine-readable presentation form of metrological data [41]. By minimum, the D-SI format requires that each numerical measurement value must be combined with the corresponding unit. The format is based on the SI unit system as it is the most commonly used unit system worldwide. However, since several other measurement units are also widely used in different applications, the format also supports the inclusion of non-SI units in addition to the corresponding SI unit. In addition to the numerical values and units, the data model enables the inclusion of relevant metadata, such as the measurement uncertainty of a measurement sample, description of the uncertainty distribution, and timestamps. An example of a data sample presented in the D-SI format is shown in Figure 1, which shows the minimum requisite metrological information in green and additional information providing a timestamp and measurement uncertainty in blue.

Similarly to the D-SI, the DCC schema is aimed to be used as a basis for a global standard for digitized calibration information [42]. The state-of-the-art processes of handling calibration data have mostly been dependent on manual work as the current calibration certificates lack machine readability. With a standardized DCC format this amount of manual work can be drastically reduced, which both reduces the time and costs required for the calibration data management while simultaneously offering a significant improvement in data integrity. Additionally machine-readability enables efficiently assessing the traceability of sensors, as the DCCs of the instruments forming the calibration chain are directly linked. Nikander et al. have presented the requirements for the secure use of DCC [4]. Standardization will be essential for establishing a framework for unambiguous and secure transfer of digital metrological data that can be used for automatized decision-making more effectively.

**Figure 2:**
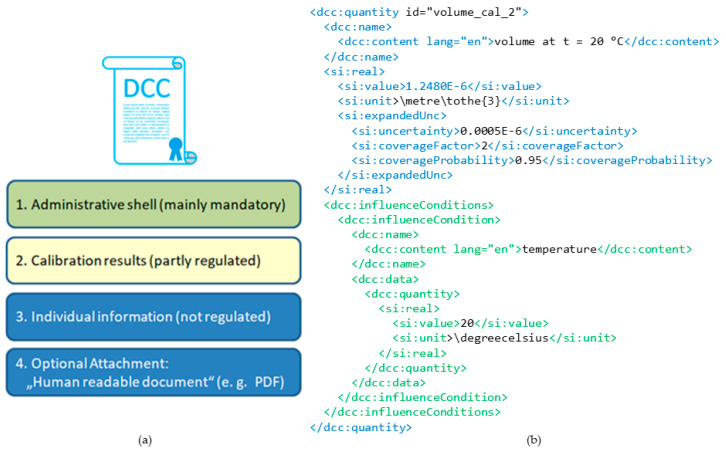
Example of the Digital Calibration Certificates (DCC) structure. High level syntax of the DCC (**a**) and an XML extract from a calibration result of a volume measurement (**b**).

The general XML structure from the DCC in the SmartCom project is outlined in Figure 2. The left side shows the top-level syntax consisting of four major parts:

- **Administrative shell:** Regulated and required information of core interest, such as a unique identifier of the DCC, calibrated items, customer, and calibration laboratory.
- **Calibration result shell:** Partly regulated area of the machine-readable measurement results for calibrated measurands, influence conditions, and relevant metadata like used measuring equipment and calibration methods.
- **Individual Information:** Non-regulated part for any additional information, such as comments, figures not relevant for the calibration result, individual domain specific data formats, etc., that are not necessarily machine-readable.
- **Optional attachment:** Data container for a typically human-readable document, such as a conventional analogue calibration certificate (e.g., in PDF format).

Figure 2 shows an example of a quantity element that is used to define data of measurement in the DCC [38]. The quantity is marked with blue. It encloses a D-SI element with a measured value of a volume. The DCC quantity extends the D-SI information with domain specific metadata, such as the name dedicated to English language and an influence condition provided with the volume measurement.

## 4. Solution Outline

As the related work suggests, there is interest and already some conceptual implementations for including the uncertainty information from sensor networks to be used, e.g., in industrial predictive ML applications. However, an overall solution on how the traceability and uncertainty information of individual sensors can be included in the systems has not been as much discussed. Availability of the forthcoming standardized formats of DCC and D-SI will enable a framework that can solve incompatibility issues and enable unified presentation for measurement uncertainty throughout whole calibration chains, all the way to individual sensors, including even low-cost IoT sensors.

Our proposed concept provides a solution for including the measurement uncertainty data, based on the calibration information of individual sensors, as metadata into the measurement data that each sensor transmits. Whether the metadata would be included to the measurement data by the sensor itself or by using a specific node (proxy) that combines the incoming data with the corresponding metadata is dependent on the application and the properties of the sensor itself.

So far, the integrity and authenticity of measurement data have not caused major problems in most applications, as the data are typically used in closed systems. However, in many cases additional benefits can be found if the data can be made available also for external use, e.g., if the data can be shared between the parties along a supply chain or, e.g., among autonomous vehicles that communicate with each other. 

It is noteworthy than in many applications (e.g., vehicles or processes in the pharmaceutical industry), the integrity and origin of the data is of utmost importance, since compromised data may lead to substantial financial loss or even life-threatening accidents. Even for accidental non-malicious compromise, it is important to be able to analyze the cause of any incident afterwards. 

In the following subsections, we outline our proposed solution, starting from securing the content of the DCCs, followed by considering the security of the measurement results from individual sensors (Section 4.2), and finally considering sensor networks (Section 4.3).

### 4.1. Digitally Signing DCCs

As a part of the ongoing work to define a security framework for DCCs, we have proposed that each DCC would be digitally signed and/or sealed. In practical terms, this involves adding an XML signature [43,44] to the DCC, both preventing the data within the DCC from alternations and proving that the DCC has been signed and/or sealed, with an identified public key, by the authorized calibration service provider. Whenever desired, such a signature or seal may be eIDAS [45] compatible, following the XAdES standard [46].

**Figure 3:**
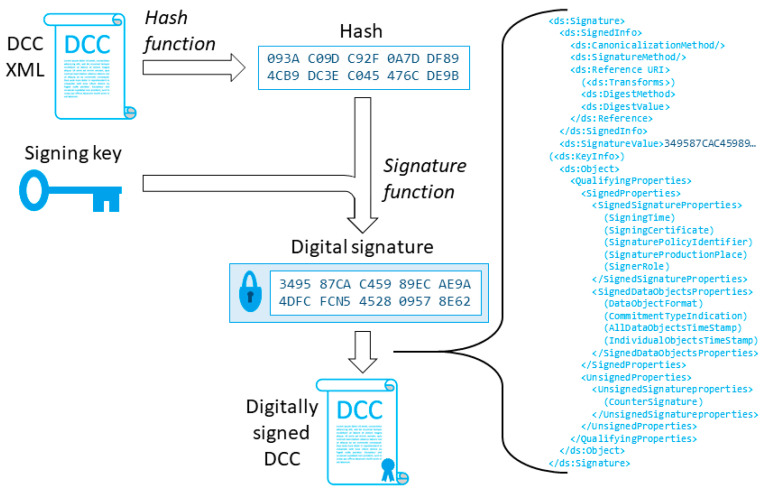
A general example of the process for digitally signing a DCC.

Figure 3 shows a general example of the process for digitally signing a DCC. First, a message digest or hash value is calculated over the DCC XML file, using a specific cryptographic hash function, such as SHA-256. Second, the private key of the person or organization issuing the DCC is used to encrypt the hash using a signature function, such as ECDSA. In XML, the signature created with the signature function may be included to the original file itself, in this case of a DCC, to create a signed and/or sealed version of the DCC. After the DCC has been signed, the signature can be verified using the public key corresponding to the private key that was used for signing/sealing.

Technically, there is no difference between a signature and a seal; both are formed in exactly the same manner. The difference is legal: according to eIDAS, a signature is formed when using a designated person’s personal key, while a seal is formed when using an organization’s key. Furthermore, it is possible to add more than one digital signatures or seals to a DCC; for example, a DCC may be first signed with a signature of a designated person and then sealed with the seal of the service provider.

### 4.2. Enhancing Measurement Data with DCCs and Digital Identifiers

In order to provide full integrity, traceability, and proof of origin for measurement results, we propose that each IoT device is provided with a digital, cryptographically strong identifier. Given the current level of technology, at this point of time, a public key from a cryptographic key pair would be most suitable for such an identifier. This is also the standard practice to secure, for example, the identity of Web servers, using RSA [47] or ECC [48] keys and X.509 certificates [49]. However, in the longer run, it appears advisable to move from static public keys into ephemeral or pseudonymic ones, in order to prevent a number of advanced attacks, including unauthorized data correlation and targeted denial of service, to name a couple. For such future practice, we propose using the hopefully forthcoming W3C Web of Things usage of device DIDs. For full privacy, we envision using Zero Knowledge [50] based DIDs.

That said, from the metrology point of view it is insufficient to be able to securely identify the devices themselves and have assurance of the device a measurement result is from. For many applications, we additionally need to know when the measurement was made, what was the known calibrated uncertainty of the device at that time, and even perhaps how the ambient conditions were at the time of the measurement.

For securing the measurement time, there are a few approaches. First, a secure time synchronization protocol may be used to provide the IoT devices with the present time [51,52]. However, the available approaches for secure time synchronization are still vulnerable to a malicious measurement timing attack, where a cunning operator makes an IoT device to use a faulty time source, as they do not as such allow secure identification of the time source. Hence, second, the secure time synchronization protocols may be enhanced to include time traceability information, for example, all the way to a known atomic clock. This is clearly an item for future research. Third, blockchains or other similar services may be used as a time stamping notary service, providing an externally signed timestamp for the measurement results.

For uncertainty and calibration traceability, we propose using the DCCs. Each DCC could include the secure digital identifier of the measurement device in its digitally signed part, thereby securely binding the DCC to the specific device. To further bind the measurement results to the DCC, each result may include a secure DCC identifier into its metadata. Such a two-way combination of the DCC and a device identifier, i.e., initially the public key from a cryptographic key pair that relates to a specific sensor, provides valuable information about the data quality, integrity, and authenticity.

### 4.3. Architectures for Traceable Sensor Networks

**Figure 4:**
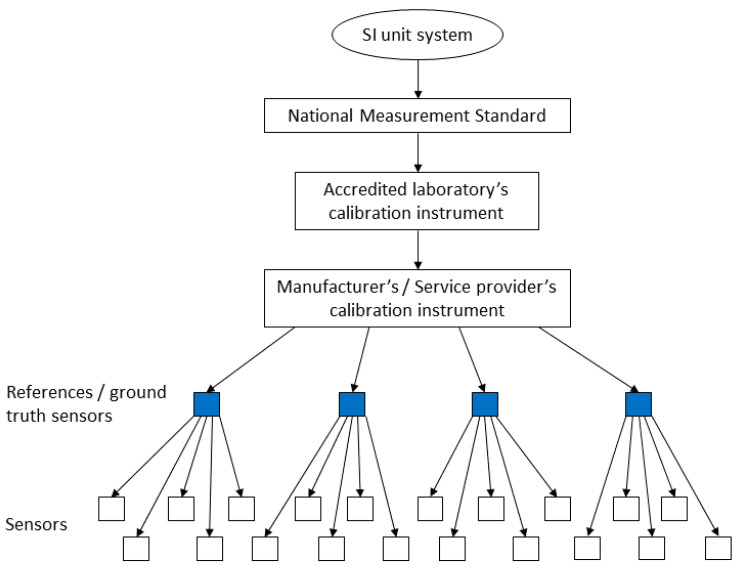
Traceability in an IoT sensor network.

Since the traceability of a measurement result is based on an unbroken chain of calibrations, ensuring the traceability of a sensor network requires a hierarchical architecture, or at least a directed acyclic graph (DAC) of the calibrations. This sets limitations for the topologies that can be considered feasible for ensuring traceability and simultaneously prevents full decentralization of the sensor network. In practice, this means that the sensor network requires a set of ground truth nodes that are used as references, and that the ground truth nodes have to be calibrated regularly with external, higher precision instruments, to minimize the measurement uncertainties resulting from the ground truth nodes being used to calibrate individual sensors. The placement of the ground truth nodes should be determined so that they would be relatively accessible, to ease the calibration procedure. Figure 4 presents an example of traceability in an IoT sensor network by displaying the calibration chain starting from the NMI’s national measurement standard and ending on the sensor level. 

Depending on the use case, the number of the ground truth nodes can be selected based on the specified tolerances for uncertainty. On the one hand, if the tolerances allow, the number of ground truth nodes can be kept relatively low and thus the physical calibration work can be minimized. On the other hand, for applications where data quality, integrity, and authenticity are most important, the optimal but simultaneously costly solution would be using only high-end sensors that are also individually calibrated regularly.

The availability of calibration data in a machine-readable format enables more precise tracking of the calibration histories of both individual sensors and sensor types. This information can be used to enhance the computational methods used for self-verification, for estimating how the measurement uncertainty of sensors will develop over their life span, and for advanced sensor prognostics.

One of the key aspects for optimizing the calibration of ground truth nodes would be minimizing interruptions in system operation. Ideally, the calibration should be executable during normal operation without any interruptions. One way to reduce the interruptions would be that the deployment positions for the ground truth reference sensors would have an additional connector for a second sensor or on-site calibration reference that would allow switching or calibrating the ground truth sensor during normal operation with minimal delays. This would require the system being flexible toward changes in sensor count and positions. This will not cause significant issues as there exists other reasons for flexible sensor count management; solutions for these kinds of issues already exist, for example, for sensor fault management [53]. Changing the sensor in a specific position would require that the deployment positions would have their own digital identifiers. Similar approach is already a common practice, e.g., in the process industry, where devices have their own identifications and position identifications separately.

**Figure 5:**
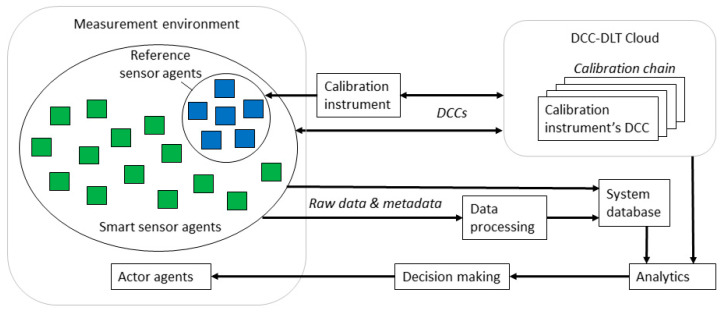
An example architecture for a sensor network system using DCC-validated measurement data.

Figure 5 presents an example architecture for a smart agent system using DCC validated measurement data. The architecture follows the principle for traceability illustrated in Figure 2, where the sensors are calibrated by the reference sensors, which in turn are calibrated using specific calibration instruments, and so on. In this architecture, the information of the whole calibration chain is archived onto a separate cloud server, which can be secured using Distributed Ledger Technology (DLT) or a blockchain to prevent manipulations. An implementation of this type of a cloud-based solution is already being investigated on European level for legal metrology [54]. Availability of the whole calibration chain information and binding the DCCs of the sensors to the data they provide can be ultimately exploited with improved analytics and decision-making. If the capabilities of the sensor agents are limited, proxies can be used for binding the metadata to the raw data.

## 5. Use Case Examples

The benefits of our proposed solution can be clarified by examining some use cases where collaborative sensing is already being applied. The use cases we have selected for this purpose are smart factories, autonomous vehicles, smart grids, and smart cities.

In general, the benefits include the possibility to prove the data quality and origin by automated validation of the DCC signatures and their respective authorities for the whole calibration chain when the DCCs are bound to the measurement data and sensor’s digital identities. This way the end user can be certain that the data are trustworthy, which opens new possibilities to use this data more openly between systems and even sharing it to external collaborators. The increased availability of the data between collaborating parties provides more extensive understanding and knowledge of large complexes, for example, in supply chain management.

### 5.1. Smart Factories

Industrial Internet of Things (IIoT) is one of the most common applications for collaborative sensing. The idea behind IIoT is that it enables an increased level of automation and better overall understanding of processes, leading to improved efficiency and thus profitability. In smart factories and manufacturing, sensor networks are used to provide data for ML applications, cyber-physical systems, and digital twins, for example, for managing manufacturing processes or supply chains [2,35,55,56]. Such applications provide frameworks for predictive analysis for maintenance, prognostics, and decision-making.

The data quality plays a significant role in the accuracy of the analyses and predictions. Better understanding of the sensors’ measurement uncertainties allows better optimization of the algorithms, for example, by weighing the data from the sensors based on the time passed from their most recent calibration, on the measurement uncertainty in that calibration, and on the individual sensor’s and sensor type’s tendency to drift. 

More trustworthy measurements are also a way to improve product quality. In industries where measurements are critical for product quality, such as precision engineering and pharmaceutical industry, the significance of the data trustworthiness is the highest. Especially in the pharmaceutical industry, autonomous systems are implemented to improve the data integrity and removing possible causes for errors [57]. In pharmaceutical industry, also the calibration of sensors is highly regulated as a part of the product quality and data integrity requirements. For these purposes alone, the introduction of DCCs would significantly streamline the calibration management processes by enabling automation.

### 5.2. Metrology-Based Metrics in Society

In proportion to the metrics used for quality management in manufacturing, metrology is used as the basis for numerous other metrics in the modern society. These metrics have been established to protect consumers and maintain critical services and infrastructures such as food production, medicine, telecommunications, and navigation [5,58]. Because of the societal significance of these application areas, the instruments and methodologies related to them are highly regulated. This area of metrology is commonly known as legal metrology. Security is an important factor in the legal metrology and since its current development is going toward more open data exchange, new technological solutions are needed to address security challenges [59].

Validation and quantification of measurement data quality, traceability, and trustworthiness are important for developing these metrics. Moreover the more accurate metrics can be established the more they can be used to promote other than economy-based aspects in societal decision-making, such as social or environmental aspects and sustainability [60,61].

### 5.3. Autonomous Vehicles

One prime example of a use case where smart sensors that can, besides the measurement data, provide the related uncertainty information while simultaneously enabling secure data exchange is autonomous vehicles [62,63]. The whole concept for safe operation of autonomous vehicles is dependent on data quality, authenticity, and integrity. In the case of independently peer-to-peer communicating vehicles, the solutions used for securing data in closed systems no longer apply, as the systems used by different manufacturers must be able to communicate safely. Thus, the need for preventing potential manipulations, e.g., man-in-the-middle attacks, increases. As earlier described, the introduction of DCCs and digital identifiers to the sensors used in autonomous vehicles would provide the capability to exchange data in peer-to-peer communication between vehicles in a secure manner.

### 5.4. Smart Grids

IoT-based smart power grid management systems are used to optimize the sufficient supply, security, and efficiency of power grids [64,65,66,67,68]. The quality and trustworthiness of the data are essential to maintain, as smart grids are critical for the society. From an emissions perspective, the significance of extensive and efficient power grids will further increase as the use of renewable energy increases in relation to the use of fossil fuels, since renewable energy sources have a higher tendency for power supply fluctuations, which means that the fluctuations in the overall power supply will increase [69]. Better knowledge of the measurement data quality and uncertainty could be used to improve the smart grid systems.

From a security perspective, power grids form a critical infrastructure in our society. Their vulnerabilities must be addressed to prevent large-scale outages. DCC validated sensors and data would provide an efficient method for securing the systems against manipulation, simultaneously providing more detailed information for sensor behavior, thereby allowing more accurate predictions for maintenance needs and prognostics.

### 5.5. Smart Cities

Smart cities are one of the common areas where the use of sensor network applications have been studied and used, which makes it a good example of how the proposed solution could benefit generic sensor network applications [70,71,72,73,74,75]. Although measurement uncertainty and data exchange security may not have such a critical role in these applications, including tools for using the sensor calibration and uncertainty information to the existing system architectures can be beneficial for data quality. This would allow developing the data processing algorithms to take into account, based on the metadata from which sensors the data are received, when that sensor has been calibrated and what the measurement uncertainty was at that time.

One approach to collecting data in urban environments is mobile crowd sensing (MCS), where consumer-centric devices such as smartphones are used collect and share data [70,71]. Since smart mobile devices have become so common, MCS enables efficient data collecting in crowded locations for monitoring, e.g., traffic and congestion. Compared to typical sensor network applications the biggest challenge in MCS is that it is based on collecting data from devices of individual users, who may prefer not to share data, e.g., due to privacy concerns. Additionally, the dependency on the users leads to somewhat compromised data integrity. Another challenge in MCS is the possibility for significant variances in data quality from different device types or models. For these reasons, the availability of information regarding measurement uncertainties and calibration status of individual devices would benefit MCS systems in identifying and weighing the data based on the measurement uncertainty. Respectively, the possibility of data integrity and origin validation increases the reliability of MCS.

## 6. Discussion

Digitalizing the calibration data enables a higher level of automation. This makes traceable calibration of sensor networks more feasible than before. Perhaps more importantly, it offers a means for diminishing the lack of traceability in the self-calibration methods of standalone sensor networks. However, the locations of the sensors and their associated ground truth nodes with calibrated reference sensors can have a significant influence on the overall applicability of the self-calibration approach. An excellent example is a network of temperature sensors filling out the whole volume of a laboratory. For example, there may be three sensors on the floor, three sensors in midair and three sensors at the ceiling. It is a natural property that you have a temperature drift from floor to ceiling and also in other directions, depending on the facility. Furthermore, it is clear that a reference sensor at the ceiling cannot be used directly to calibrate a sensor on the floor, as you always have the systematic temperature drift from the different positions. Hence, correction of such systematic deviations may play an important role for self-calibration in sensor networks, too. Measures against this problem could be: (a) a denser distribution of reference sensors, (b) the ability to manually change the locations of reference sensors, or (c) at certain intervals putting all sensors into a controlled environment with almost equal ambient conditions and performing the calibration.

### 6.1. Remaining Challenges and Future Work 

There are still several challenges in global digitalization of metrology and its infrastructure, before introducing DCC-based data exchange systems, such as our presented concept, are totally viable in real industrial applications. First, since metrology is applied in a vast amount of industries and the applications in an industry may be subject to various regulations, redefining all of the regulations to support digitalized metrological data will be a challenge. Ideally, the first steps for introducing DCC-validated data to the systems would be through de-facto standards, if a reasonable number of operators and service providers per industry are willing to commit into advancing the initialization and development of the technologies. Once the regulations are updated, the benefits of the digitalized metrology can finally be brought to the most extensively regulated and critical systems. For these reasons, updating the entire infrastructure to support DCCs will take time. 

Second, the measuring of different physical quantities is based on a wide range of technologies. The upcoming standards must eventually be applicable for all of them. Additionally, even if the technologies used are similar, the current system level implementations are not standardized. This means that attention to defining ontologies corresponding to each quantity will be essential for the applicability of the future standards for the DCC and D-SI.

The current work in the EMPIR project SmartCom provides a basis for the standardization process, but to resolve the aforementioned challenges regarding metrology, the work started in SmartCom needs to be extended. In the case of the D-SI data model, the development work aiming for eventual standardization will be continued in the CIPM Task Group on the Digital SI [76]. For the work regarding DCCs and the rest of the infrastructure, a follow-up project for SmartCom has been proposed [77]. 

### 6.2. International Applicability

One remaining challenge is the secure provisioning of the DCCs. Today, the paper calibration certificates are signed with a pen and distributed by mail or by email as PDFs, typically between organizations and people that have a long business relationship and who trust each other. As a result of the human-to-human relationships, fraud is extremely rare. On the legal metrology side, where a merchant may have an incentive to manipulate their grocery scale or petroleum pump for their own benefit, there is more fraud, and that remains a problem in many developing countries (cf., e.g., [78]). However, when going fully digital and removing the human-to-human relationship from the loop, we should expect a much higher level of fraud, unless measures are taken to prevent it.

Consequently, probably the main remaining security challenge is to provision a means for being able to verify the digital signatures in DCCs. In practical terms, to verify a digital signature, the verifier needs to know for whom the associated public key belongs to. If the verifier cannot be sure that the public key belongs to a trusted party, the signature is worthless, because anyone could have created the associated key pair and created the signature.

The usual way of associating public keys with trusted parties is to use a certificate authority infrastructure, aka a Public Key Infrastructure (PKI). For the Web, almost everyone uses the Internet X.509 Public Key Infrastructure [79]. However, while the Internet PKI is considered secure enough for a number of purposes, including for example electronic commerce, it is not considered, as such, secure enough for banking or e-health services. In the case of banking, for example, the banks require additional credentials, such as one-time passwords or two-factor authentication, in addition to the baseline session security provided by the TLS, which in turn is secured by the Internet PKI. 

Considering that fraud with measures may result in even loss of life, we do not consider the Internet PKI secure enough for the most demanding sensor solutions, such as those in many industries. For at least the most demanding use cases, something more secure is needed. In a word, we surmise that in the long term the right choice is to create a new PKI for metrology, perhaps using the most modern technologies, including DLTs for persistence and DIDs for privacy. From our point of view, such a PKI might well be feasible, especially as the BIPM, NMIs, and NABs would form a natural, already highly trusted layer for needed security certificate authorities (CAs). However, reaching such a PKI is probably years away, as it may require organizational changes and substantial security competence development in many of the organizations involved. 

That said, in Europe it may be sufficient to rely on the eIDAS regulation and the nationally managed infrastructures for secure digital identifiers of the citizens and companies. We are currently in the process of evaluating the feasibility of such a solution. However, a major drawback of an eIDAS-based solution would be its geographic applicability, since it would be usable only in the EU countries and the few others that have decided to have eIDAS compatible national infrastructures.

## 7. Conclusions

Collaborative sensor networks can be used for gathering large amounts of data cost-efficiently. One major problem with sensor networks has been poor data quality and trustworthiness. That is why various data processing methods are used to make the available data more usable. However, in terms of metrology these data processing methods cannot provide traceability to the measurement standards that are maintained by the National Metrology Institutes (NMIs) to ensure global comparability of measurement results. Traditional methods for ensuring this traceability have not been feasible and/or cost-effective for many sensor network applications. As a result of this, the quality of data has often been somewhat compromised. Similarly, the security of the IoT sensors and the trustworthiness of the data they provide have been relatively poor, as in many cases the additional costs that would have resulted from security solutions have been minimized.

Currently, metrology is going through a transition toward digitized data formats, as Digital Calibration Certificates (DCCs) are being developed as a substitute for traditional paper-based calibration certificates, and Digital SI is being developed to provide a universal data model for metrological data. Availability of digitized metrological data and calibration information will improve the applicability of traceable calibrations, also for sensor networks. Respectively, cryptographic digital identifiers and Distributed Ledger Technologies (DLT) are being developed to solve the issues related to security and trustworthiness.

Our proposed solution, which is based on introducing DCCs, D-SI, and cryptographic digital identifiers to the sensors and collaborative sensor network applications, would provide the means to bring measurement metadata available to the systems, thereby increasing the significance and trustworthiness of the data. The largest benefits of DCC validated data can be found in applications where data quality and trustworthiness are critical for the process, such as in the manufacturing systems in pharmaceutical industry and in data sharing between autonomous vehicles.

However, a large-scale implementation of the solution is not yet feasible, as there is still work required to be done on the technologies and the corresponding infrastructures, before they can be standardized globally. Yet, the issues these technologies address are so significant that a number of research projects covering these topics are taking place and additional ones are being proposed with the aim of establishing the infrastructure needed for the digital transformation of metrology and developing prototype implementations allowing more substantial evaluation of the benefits in different industries [54,77,80,81,82].
